# A qualitative study of health workers’ perspectives on malaria case identification and management among pregnant women in Savelugu Municipality, Ghana

**DOI:** 10.1371/journal.pgph.0001963

**Published:** 2023-05-24

**Authors:** Martin Nyaaba Adokiya, Michael Boah, Solomon Abotiba Atinbire, Felix Achana, Joyce Aputere Ndago, David Abatanie Kanligi, Zakaria Abotiyire, Cheryl A. Moyer

**Affiliations:** 1 Department of Epidemiology, Biostatistics and Disease Control, School of Public Health, University for Development Studies, Tamale, Ghana; 2 Holy Cross Catholic Church, Tamale, Northern Region, Ghana; 3 Department of Social and Behavioral Change, School of Public Health, University for Development Studies, Tamale, Ghana; 4 Pediatric Unit, Savelugu Municipal Hospital, Ghana Health Service, Northern Region, Ghana; 5 Department of Learning Health Sciences and OB/GYN, University of Michigan, Ann Arbor, MI, United States of America; PLOS: Public Library of Science, UNITED STATES

## Abstract

Despite successes in malaria control interventions over the past two decades, malaria remains a major public health concern. Over 125 million women live in endemic areas and experience adverse pregnancy outcomes due to malaria. Understanding health workers’ perspectives on malaria identification and management is important to informing policy changes on the control and eradication of the disease. This study explored the perspectives of health workers on malaria case identification and management among pregnant women in Savelugu Municipality, Ghana. A qualitative study with a phenomenology design was conducted among participants. Participants were purposively selected and interviewed using a semi-structured interview guide. Thematic analysis was performed and the results were presented as themes and sub-themes. Four themes and eight sub-themes regarding case identification and management of malaria in pregnancy were identified including malaria case identification training (trained and untrained), identification approach (signs/symptoms and routine laboratory test), diagnostic tools (rapid diagnostic test and microscopy) and management options. It revealed that attending malaria training programs was generally optional. Some of the participants had not undergone any refresher training for malaria identification after their formal training at health institutions. Participants identified malaria by its signs and symptoms. However, they often referred clients for routine laboratory tests for confirmation. When malaria is confirmed in pregnancy, quinine is used for first trimester treatment, while Artemisinin-based Combination Therapies are prescribed after the first trimester. Clindamycin was not used in the first trimester treatment. This study found that training programs were optional for health workers. Some participants have not received refresher training after graduating from health institutions. Treatment of confirmed cases did not include clindamycin for first trimester malaria infections. Malaria refresher training programs should be made mandatory for health workers. Every suspected case should be confirmed using Rapid Diagnostic Test or microscopy before treatment.

## Introduction

In the past two decades, there have been successes in malaria control efforts. However, current malaria statistics show that progress has stalled due to the complexity of issues including low investment, reduction in vector control interventions, and inadequate access to quality health care since 2015 [1]. Sub-Saharan Africa (SSA) bears the greatest burden of malaria infection with over 70% of all global cases [2]. Additionally, SSA contributes about 98% of malaria mortalities and it has 10 of the 11 most endemic malaria countries in the world [1, 2]. In SSA, the high burden of malaria requires renewed efforts. This includes enhanced political commitment, increased funding and strengthening of malaria workforce capacity [1].

Pregnancy increases the vulnerability of women, makes them susceptible to infections. It is estimated that 125 million women live in malaria-endemic areas and experience severe malaria infection during pregnancy [3]. Malaria in pregnancy can contribute to adverse birth outcomes, including stillbirth, spontaneous abortion, fetal growth retardation and low birth weight [4–6]. Malaria infection thus poses danger significant risk to both mothers and their unborn babies. Therefore, interventions to reduce malaria-related complications in pregnancy should be geared towards early detection and prompt management of confirmed cases.

The World Health Organization (WHO) has recommended three approaches for managing malaria in pregnancy; effective use of insecticide treated nets (ITNs), intermittent preventive therapy with sulphadoxine pyrimethamine (IPT-SP) and management of confirmed cases with artemisinin-based combination therapy (ACT) or quinine [7]. The gestational age of pregnancy influences the type of treatment prescribed for a positive malaria infection. Quinine and clindamycin are used in the first trimester of pregnancy, while ACTs are used in the second and third trimesters of malaria infections [7, 8]. However, issues of “over prescription” of anti-malarial medications emanating from presumptive diagnosis warranted the “test before treatment” policy [9]. Consequently, health workers are mandated to confirm a positive malaria test before initiating treatment. In addition, growing resistance to IPT-SP required alternative interventions for malaria management among pregnant women. A suitable option with promising results was the intermittent screening and treatment (IST) strategy [10, 11]. With this strategy, pregnant women are screened periodically for malaria and those who tested positive are treated with dihydroartemisinin-piperaquine instead of the prophylactic IPT-SPs.

Regardless of the treatment approach, early case detection and prompt management of malaria in pregnancy are essential to averting complications. Hence, ongoing screening of pregnant women, identification, and management of malaria should be the priority of the malaria workforce. Rapid diagnostic tests (RDTs) are cost-effective, simple and accessible malaria screening tools, yet they are inefficient for low density parasite densities [12, 13]. Follow up testing for malaria parasites with microscopy becomes applicable in instances where RDTs are deemed unreliable [14].

In this context, there is inadequate capacity of malaria workforce across SSA, and new approaches may need consideration [15]. Hence, this study explored the experience of health workers on malaria case identification and management among pregnant women in Savelugu Municipality, Ghana, in an effort to better understand both challenges and opportunities to improve outcomes.

## Materials and methods

### Study design

This paper reports the findings of a phenomenological study involving in-depth qualitative interviews among 13 health workers who provide antenal care (ANC) or screen for malaria among pregnant women. The study explored their perspectives on case identification and management of malaria in pregnancy. The conduct of the study and analysis of the data followed a phenomenological thematic approach as proposed in previous studies [16]. The study was conducted between July and December, 2019.

### Study setting and participants

The study was conducted in four health facilities within the Savelugu Municipality, namely; the Savelugu Municipal Hospital, Savelugu Reproductive and Child Health Center, Moglaa Health Center and a private facility (Mbia Laboratory). The participants were purposively selected from these facilities based on their roles in the case identification and management of malaria among pregnant women, as well as their willingness to participate in the study. Participants were approached through a written invitation and asked about their interest in participating in the study. Each was taken through an informed consent document prior to beginning the interviews, which were conducted in a separate area at the health facilities. The study participants consisted of nurses, midwives, medical doctors, and laboratory professionals (Table 1). To ensure consistency, all interviews were conducted by the same researcher, who is trained in qualitative interviewing techniques.

**Table 1:** Sociodemographic characteristics of study participants.

S/N	Gender	Role	Work experience (years)
1	Male	Senior Staff Nurse/ Facility In-Charge	2
2	Male	Senior Staff Nurse	6
3	Female	Staff Nurse	2
4	Female	Enroll Nurse	4
5	Male	Enroll Nurse	4/12
6	Male	Community Health Nurse/ Sub-District Head	4
7	Female	Principal Midwifery Officer /Ward In-Charge	5
8	Female	Midwifery Officer /Ward In-Charge	6
9	Female	Senior Staff Midwife	2/12
10	Female	Midwifery Officer	1
11	Female	Laboratory Technician	7
12	Female	Principal Biomedical Scientist/ Lab Manager	9
13	Male	Medical Officer	1

### Data collection

A study-specific semi-structured interview guide was developed using an iterative development process amongst the investigators. This involved using study objectives to draft potential questions, and then circulating draft questions amongst the researchers to be refined and revised before being pilot tested.

The interview guide commenced with general questions about providers’ experience in healthcare, followed by questions related to experiences in identifying, reporting and treating malaria among pregnant women. Probes such as, ‘Can you explain?’ or ‘Please can you give an example?’ were asked to clarify the perspectives of participants. Each interview took about 45 minutes to complete.

Sampling was discontinued when the data reached saturation; which is when no new codes could be formed in subsequent interviews [17]. This was realised after the 10^th^ interview, following regular discussions amongst the researchers and the ongoing development of a potential list of codes. Nonetheless, three more interviews were conducted to ensure that no new codes emerged. All interviews were conducted face-to-face and audio recorded (using tape recorder) with the consent of the participants.

### Data analysis

Data analysis followed the thematic framework approached as described in previous studies [16, 18]. Audio recordings were transcribed verbatim and read repeatedly by two of the research team (DAK and MNA). The texts were read word-by-word and initial codes were identified and discussed as a team. Coding was then conducted by (DAK and MNA) line-by-line using comments in Microsoft Word. The codes were then organized into related categories, forming broad themes that reflected patterns in the data and were relevant to the research objective. The themes were reviewed and checked to determine if they reflected the context of the source data. The themes were redefined and organized into related meaningful wholeness leading to the formation of main themes and sub-themes. The analysis process was routinely discussed and revisited by all authors.

### Rigor of findings

We adopted Lincoln and Guba’s criteria to ensure the study was scientifically sound: credibility, dependability, confirmability, transferability and authenticity of findings [19]. Credibility of the findings was achieved by ensuring prolonged engagement with the data, member checking and peer debriefing. The final codes were presented to two of the participants to confirm that their viewpoints were captured. This was done as a way to ensure member verification. For the purpose of peer checking, the researchers invited three experts in qualitative research to validate the codes and data analysis process. To enhance the dependability of the results, the methods of coding were comprehensively described, and the participants’ perspectives were presented as thick and rich text. Codes drawn from the interviews were also sent back to participants for verification as a way of ensuring veracity of the information. Corrections were made to the initial codes to reflect participant perspectives when necessary. Transferability of findings was achieved by ensuring that the process of participant selection, methods of data collection and analysis as well as documentation of participants’ experiences were reflective of the study objective.

### Ethics consideration

The study was reviewed and approved by the Ghana Health Service Ethics Review Committee (**GHS-ERC 017/06/19**). The objective of the study was explained to the participants and they decided the time convenient for each interview. Each participant signed a written informed consent form and was assured they could withdraw from the study at any point without consequences. Participants were also assured of the anonymity and confidentiality of the information they provided.

## Results

A total of 13 interviews were conducted among different categories of health workers. They included six (6) nurses, four (4) midwives, two (2) laboratory workers, and one (1) medical officer. Among the participants, five (5) were males and eight (8) were females. The participants also had an average work experience of 3.6 years (ranging from 2 months to 9 years) (Table 1).

### Themes and sub-themes

The study explored the experiences of health workers regarding malaria case identification and management among pregnant women seeking care at health facilities within Savelugu Municipality. The study identified four (4) main themes and eight (8) sub-themes. The main themes of the study included; malaria case identification, training, a case identification approach, diagnostic tools and management options (Table 2).

**Table 2:** Main themes and sub-themes of the study.

Theme	Sub-theme
Malaria case identification training	Trained
	Untrained
Case identification approaches	Signs and symptoms
	Routine laboratory test
Diagnostic tools	Rapid diagnostic test (RDT)
	Microscopy
Management options	First trimesters treatment
	Second/ third trimester treatment

### Malaria case identification training

The study explored training opportunities for health workers on malaria identification. Some insights were generated from the participants, including sub-themes in both trained and untrained categories.

#### Trained

From the participants’ perspectives, they had opportunities to participate in refresher training on malaria case identification among pregnant women. Some of the participants maintained that the training sessions were generally voluntary and not obligatory for the staff.

*‘‘I think they organized one training here with the Staff of the Savelugu hospital*. *Some of us participated because it was voluntary for those who were actually willing*. *That was when they changed the method of*, *instead of using the + one method in 2018 (*Parasite densities: + = 10 to 100 parasites/ul; ++ = 100 to 1,000 parasites/ul; +++ = 1,000 to 10,000 parasites/ul; ++++ = > 10,000 parasites/ul*)*, *we were advised to use the count method (*count the number of parasites in Red Blood Cells*)”* **(Laboratory Technician).**

*‘‘Yes*. *I participated in a training program in 2015*, *I was at Builsa South District by then*. *It was the National Malaria Control Program that organized training for midwives at the Xtee Crystal Hotel in Bolgatanga*. *During the SP (sulphadoxin pyrimethamine) validation at the training*, *we were taken through malaria case identification among pregnant women*. *We were shown how to conduct the rapid diagnostic test for malaria identification”* (**Midwifery Officer).**

Some participants had the opportunity to participate in more than one training sessions.

*‘‘Yes*. *I was trained twice somewhere in 2011 and the recent one was in 2019*. *We were trained to also train some staff in the district*. *It was organized by the Ghana Health Service under the National Malaria Control Program in Tamale”* **(Principal Biomedical Scientist).**

#### Untrained

Some staff who managed malaria cases were not trained on case identification of the condition within health facilities in the Savelugu Municipality. The participants reported that they had to apply knowledge gained during their training at health institutions to identify malaria cases among pregnant women.

*‘‘No*. *I have not received any specific training on malaria case identification*. *I have personal knowledge of malaria*. *When I see you and I know that you are pale and the way you look*, *it may be some sign of fever or malaria in you*. *I will recommend for the pregnant woman to be tested for malaria”* **(Enroll Nurse).**

*‘‘No*. *I have not been trained but my colleague has received the training*. *She is part of the malaria control program*. *I don’t know how many times a year*, *but she has been going for the training*. *Because we are two medical officers in charge of the units*, *both of us cannot leave the facility at the same time*. *She is specifically in charge of the malaria training*. *The last training was just about two months ago*. *I think it was at the Regional Health Directorate or the Tamale Teaching Hospital*, *I am not too sure*. *I have knowledge from what we were taught at medical school*, *and when my colleagues come from the training*, *they also brief us”* **(Medical Officer).**

### Case identification approaches

The participants also described their experiences with malaria case identification approaches among pregnant women. They identified malaria from the signs and symptoms the pregnant women presented or by conducting routine laboratory tests.

#### Signs and symptoms

From the participants’ perspectives, the commonest approach to identifying malaria among pregnant women is through the complaints they present during the care process. Health workers are able to identify malaria symptoms by interacting with their clients. Based on these signs and symptoms, the pregnant woman maybe referred for laboratory tests.

*‘‘After counselling and palpating during antenatal care*, *the client is asked some questions and based on the responses that will be provided and if the signs/symptoms (fever*, *bodily pains etc*.*) indicate malaria*, *we will refer them with a note to the out-patient department (OPD) where rapid diagnosis test (RDT) is used to test”* **(Midwifery Officer).**

*‘‘The clinical signs such as fever*, *vomiting*, *headaches*, *chills*, *loss of appetite and the rest are there*. *Apart from that*, *we refer them to OPD for RDT to be done*. *We refer them to the laboratory too*, *but I can’t tell what goes on in the laboratory*. *We only send them and they come with the results”* **(Community Health Nurse).**

*‘‘…we pick it from the signs and symptoms that they will give us*. *And when they give those signs and symptoms*, *then we will then ask them to go the clinician*. *At the clinician’s place*, *they will also assess whatever the pregnant woman presents*. *If they want the pregnant woman to do any malaria test*, *then it is performed and the treatment be administered from there”* **(Principal Midwifery Officer).**

*‘‘…but when we see the person*, *we just look at her face and know that this person is suffering from either anemia or she is suffering malaria*. *But the right thing is that we write a request form for the person to go for the test because we don’t conduct the test at this facility*. *When the person comes and it is positive*, *then we will declare the person at once to have malaria”* **(Enroll Nurse).**

For a health prescriber, the first indication for laboratory investigations is the signs and symptoms that the pregnant woman presents:

*‘‘This one*, *just like any other patient*, *we usually use the symptoms and signs*. *If these correlate to malaria infections*, *we will let them go to conduct the laboratory test*.*…*. *So basically*, *it is the signs and symptoms*. *The person comes with the symptoms*, *then we will ask for the signs”* **(Medical Officer).**

#### Routine laboratory test

Nurses and medical doctors primarily depend on signs and symptoms to identify malaria cases. However, their colleagues working at the laboratory rely solely on laboratory tests. They explained that two basic procedures are conducted to identify malaria in the laboratory; the Rapid Diagnostic Test (RDT) and microscopy.

*‘‘So basically*, *two methods are used to identify malaria; the RDT and Slime methods*. *This is similar to microscopy*. *The RDT method*, *which is like a cassette*, *where a buffer is provided with an alcohol pad*. *The thumb of the patient is cleaned and pricked*. *Some blood is applied to the specific place made for the sample*. *The buffer is added in one or two drops depending on the nature of the sample*. *When the blood is anemic*, *you do not add buffer*. *So*, *it might run the test invalid*. *For the slime method*, *samples taken are run on the slide and allowed to dry for a few minutes then you stain*, *wash*, *and allow it to dry for microscopic examination”* **(Lab Technician).**

*‘‘We use microscopy and at times RDTs when it is available*. *However*, *most of the time*, *it is* microscopy” **(Principal Biomedical Scientist).**

### Diagnostic tools

With regards to the specific tools used to identify malaria cases among pregnant women, the participants reported using two tools—RDT and microscopy. The choice of tool was influenced by the type of healthcare setting. Health workers in the laboratory had access to both RDT and microscopy, while their colleagues at the wards used only RDT to diagnose malaria.

#### Rapid diagnostic test (RDT)

According to the participants, the RDT is the initial test and it is conducted on pregnant women when malaria is suspected. The prevailing signs and symptoms usually influence the decision to conduct an RDT. They further explained that RDT is quicker to perform and that treatment can be initiated early for pregnant women who test positive.

*‘‘…It is still the RDTs*. *When we take their history and suspect malaria in the pregnant women*, *we do our assessment*, *run our RDTs and if it is not positive*, *we refer them for the microscopy test”* **(Staff Nurse).**

‘‘…*It is the same like the one I mentioned*. *We do not want to delay treatment for malaria because it is an endemic disease in the community*. *The hospital has made the investigation a bit faster through testing at the OPD level*. *So as soon as pregnant women come in and have those symptoms*, *they are tested at the OPD even before they go to see the doctor*, *except when there are no kits*. *So*, *it is the same RDT too for pregnant or non-pregnant women and children”* **(Medical Officer).**

*‘‘It depends on the complaints they present*. *Based on the complaints and signs and symptoms they present*, *we conduct the RDT test to identify it [malaria]*. *So*, *if it is positive*, *then we will continue from there with treatment*. *However*, *if it is negative*, *we usually advise them to sleep under mosquito nets*. *In addition*, *when pregnant women are due for the IPT [intermittent preventive treatment]*, *we administer it to them”* **(Staff Midwife).**

#### Use of microscopy

Microscopy is an option available to only laboratory workers. It is mostly a follow-up test of the RDT to confirm the presence of the malaria parasite in the blood of pregnant women.

*‘‘The Slime method is the same as microscopy*. *The samples are run on the slide*. *It is allowed to dry for a few minutes and then you stained*. *After the staining*, *the normal washing is performed and allowed to dry for microscopic examination”* **(Lab Technician).**

### Management options

The participants reported that treatment options for malaria in pregnant women are influenced by the gestational age of the pregnancy. They emphasized that, quinine or artemisinin-based combination therapy (ACT) is used to treat malaria in pregnancy.

*‘‘It depends on the gestational age of the pregnancy and the severity of the malaria*. *Every pregnant woman comes with her own case*, *complication*, *and gestational age*. *So*, *what the health workers do is if you are positive for malaria*, *they will check for the gestational age of the pregnancy*. *This is used to determine the type of drug to be used”* **(Enroll Nurse).**

#### First trimester treatment

Participants reported that women with less than three months of pregnancy (first trimester) are treated with quinine when they are infected with malaria. Depending on the severity of the malaria, either oral or injection formulations are served.

*‘‘From the current standard or protocol*, *the health managers insist that for uncomplicated malaria we should go with quinine tablets for the first trimester of pregnancy*. *However*, *except that most of the pregnant women from our experience*, *they bleed when we start the treatment with the quinine*. *For severe malaria in pregnancy*, *we give them IV quinine in the first trimester”* (**Medical Officer**).

*‘‘…so if a pregnant woman comes in and she is tested positive*, *and the gestational age is between one week to and quickening (that is in the 1*^*st*^ *trimester)*, *we usually give the quinine tablets because it is uncomplicated malaria”* **(Senior Staff Nurse).**

#### Second/third trimester treatment

According to the participants, Artemisinin-based Combination Therapy (ACT) is used to treat malaria in pregnancy after the first trimester of gestation.

*‘‘When the pregnant woman experiences quickening*, *we do not give quinine for a positive test*. *However*, *we put the person on ACT; Artesunate Amodiaquine and Artemether-Lumefantrine*. *Therefore*, *the appropriate treatment depends on the gestational age of the pregnancy”* **(Staff Nurse).**

‘‘*If the pregnancy is above the second and third trimesters*, *the health workers may use Artesunate Amodiaquine and Artemether-Lumefantrine for treatment”* **(Principal Midwifery Officer).**

*‘‘For the second and third trimesters*, *the health workers use either Artemether-Lumefantrine or Artesunate-Amodiaquine tablets for the treatment of malaria*. *However*, *if the malaria is a complicated one*, *we use the artemether injection for treatment”* **(Midwifery Officer).**

## Discussion

This study explored the experiences of health workers on malaria case identification and management among pregnant women attending antenatal care at selected health facilities in Savelugu Municipality of Northern Ghana. In all, four main themes were identified; malaria identification training, identification approaches, diagnostic tools, and management options.

The results revealed that some of the health workers had not received refresher training for malaria case identification in Savelugu Municipality. Participants recounted using personal experience or depended on knowledge gained during their pre-service at health training institutions to identify malaria in pregnancy. Similar findings of inadequate training of care providers were reported in China and Uganda [20, 21]. Whereas in China, low capacity of health workers on malaria identification could be explained on the basis of low exposure to malaria cases, the situation in Africa is different. China is on course for malaria elimination and eradication. This implies reduced exposure, low political commitment, and decreased investment in the malaria workforce [20]. However, malaria remains endemic in SSA. Thus, building the capacity of health workers is essential for malaria control and elimination efforts [15]. Early detection of malaria is particularly crucial for effective management and the prevention of complications in pregnancy. Thus, training programs for malaria identification should not be voluntary for health workers. Contrary to expectations, our study identified lapses in this area. The health workers reported that attending malaria training programs was optional. However, on-the-job training and capacity strengthening are supposed to be obligatory for all malaria workforce [15]. The current finding requires urgent attention. It is a pre-requisite to ensure regular malaria identification training as part of measures to controlling the infection within endemic and vulnerable populations. Thus, the enforcement of regular refresher training for health workers may contribute to the efficient management of malaria cases in sub-Saharan Africa.

This study also showed that health workers adopted the signs and symptoms or routine laboratory testing approach to screening for malaria among pregnant women in Savelugu Municipality. The participants explained that their skills for identifying malaria in pregnant women emanated from training sessions or experiences gained from managing malaria cases previously. After a preliminary diagnosis or assessment of malaria infection, the suspected cases may be referred for laboratory testing and confirmation. The current finding corroborated that of a study conducted in Malawi, where health workers relied on signs and symptoms as a screening tool for malaria infection [22]. Though this approach may be helpful, asymptomatic malaria would largely be missed. Hence, the use of an intermittent screening and treatment (IST) strategy can be an effective synergy for the early identification of malaria among pregnant women [5, 10].

In addition, this study revealed that health workers used RDT and microscopy as tools for identifying the presence of malaria parasites in the blood of pregnant women. Particularly, nurses, midwives, and a medical doctor reported using RDTs to diagnose malaria in pregnancy, while the laboratory staff used microscopy as an additional tool. This finding is consistent with the general practice of malaria case identification in the Ghanaian healthcare system [23–25]. Routine use of RDTs is a quick, cost-effective, and an easy-to-use strategy for malaria case identification. In addition, it does not require specialized training to operate. Thus, it is efficient in resource limited settings [13, 26]. Generally, health workers comply with testing protocols for malaria screening due to the user-friendly nature of RDTs in SSA [22]. However, irregular supply and issues of poor sensitivity (due to low malaria parasite density) make RDT inefficient for identifying malaria parasites particularly in asymptomatic patients [14, 27]. Therefore, it may require additional microscopy evaluation to help identify malaria when RDT is deficient.

This study also explored the experiences of health workers regarding management options for malaria in pregnant women. The participants explained that treatment of malaria in pregnancy depended on the gestational age. Quinine is prescribed for the first trimester, while artemisinin-based combination treatments (ACTs) are the preferred drugs for the second and third trimesters. Consistent with a similar study in Burkina Faso, health workers treated malaria in pregnancy with an ACT [28]. However, the previous study did not specify the gestational age of the pregnancy, unlike our study. Studies from India also reported that health workers adopted unorthodox methods for malaria prevention and treatment [29]. The recommended treatment regimen for uncomplicated malaria in the first trimester and second/third trimesters of pregnancy is oral quinine with clindamycin and ACTs respectively [7, 8]. In severe infections, parenteral formulations may be prescribed for malaria treatment. The use of ACTs in the second and third trimesters of pregnancy is deemed safe and efficacious [5, 27]. Although there is inadequate data to ascertain the safety profile of ACTs, their use in the first trimester cannot be ruled out entirely [30]. Thus, our finding is consistent with the general recommendation for antimalaria therapy in pregnancy. However, the use of clindamycin in the first trimester was not reported in our study. There is the potential for inconsistent findings with regards to antimalarial therapy among pregnant women [28, 29]. Recent reviews suggest that this could be due to lapses with national malaria treatment guidelines [8]. Therefore, there is a need for further studies to ascertain the use of clindamycin and quinine for malaria treatment in the first trimester within our study setting and beyond. An evaluation of the current national malaria treatment guidelines may also help to ascertain discrepancies with the WHO’s updated versions [8].

## Strengths and limitations

This study has some strengths. It is the first study to explore health workers’ experiences with malaria case identification and management in the Savelugu Municipality. The study design was also flexible, which allowed the researchers to purposively select the study participants and explored their experiences with malaria case identification and management.

The study also has limitations. It was an explorative study with a small sample size. Findings are subjective to participants’ experiences. Hence, the findings of this study should be interpreted with caution as they may not be applicable in other settings. The use of clindamycin in the first trimester of pregnancy could not be established in this study. Future studies may consider these limitations in their designs.

### Conclusion

In this study, participating in malaria training programs was reported to be optional for health workers in the Savelugu Municipality. Some of the health workers had not undergone any refresher training for malaria identification after their formal training. Additionally, health workers identified malaria by its signs and symptoms. Mostly, clients are referred for routine laboratory tests and confirmation. Quinine and ACT are used to treat malaria in the first trimester and second/trimesters respectively. There is a need for health facility managers to ensure malaria training programs are mandatory for staff. Further studies should be conducted to evaluate the national malaria treatment guidelines in light of WHO’s updated versions.

## List of abbreviations

ACT: artemisinin-based combination therapy
ANC: antenatal clinic
IPT: intermittent preventive therapy
IST: intermittent screening and treatment
ITN: insecticide treated net
OPD: out-patient department
RDT: Rapid diagnostic test
SP: Sulphadoxine pyrimethamine
WHO: World Health Organization
